# Full-Depth Dynamic Gradient-Guided Residual Correction for Daily Three-Dimensional Ocean Sound Speed Prediction

**DOI:** 10.3390/s26154939

**Published:** 2026-08-04

**Authors:** Jiachen Yang, Qing Xie, Zhengjian Li, Desheng Chen, Changlin Chen, Jiabao Wen

**Affiliations:** 1 School of Electrical and Information Engineering, Tianjin University, Tianjin 300072, China; 2 Institute of Geographic Sciences and Natural Resources Research, Chinese Academy of Sciences, Beijing 100101, China

**Keywords:** 3D ocean sound speed prediction, residual correction, temperature gradient, ConvLSTM, full-depth dynamic mask, acoustic propagation

## Abstract

Accurate daily three-dimensional (3D) ocean sound speed prediction is essential for underwater acoustic applications. Existing data-driven studies often focus on global field errors, leaving localized thermocline-related errors insufficiently addressed. Using ten years of daily GLORYS12 reanalysis data from an equatorial Pacific region, we developed a 3D ConvLSTM predictor for background spatiotemporal evolution. We then proposed the Full-Depth Dynamic Gradient-Guided Residual Correction Network (FDGRC-Net), which derives a continuous mask from the previous-day regional temperature gradient profile to guide residual learning and output gating. On the 2009 test set, FDGRC-Net reduced the overall RMSE from 0.4209 to 0.3916 m/s and the MAE from 0.2302 to 0.2180 m/s. It also demonstrated improved prediction performance in the northern South China Sea cross-region evaluation and achieved annual RMSE reductions of 5.12–7.90% in frozen 2010–2015 tests. Against 521 collocated Argo profiles, it slightly but consistently improved the observational agreement. In a controlled Bellhop diagnostic, the transmission loss MAE decreased from 2.825 to 2.489 dB and the mean arrival time error from 4.099 to 3.653 ms. These results demonstrate that FDGRC-Net provides an effective and physically interpretable approach to thermocline-aware daily 3D sound speed prediction.

## 1. Introduction

The accurate acquisition and prediction of 3D ocean sound speed fields are essential for underwater acoustic applications in complex marine environments [1,2,3,4]. Reliable environmental characterization is also relevant to biologically inspired covert underwater communication, including dolphin sound micro-modems and humpback whale-like bionic Morse schemes [5,6,7,8,9,10]. Traditionally, the sound speed is calculated from temperature, salinity, and pressure using empirical equations or equation-of-state algorithms [11,12]. However, in situ temperature and salinity profiles are often sparse in both space and time, making it difficult to provide continuous daily regional 3D fields. The availability of Argo observations and eddy-resolving ocean reanalysis products has therefore encouraged a transition from isolated profile estimation toward the data-driven reconstruction and forecasting of spatially continuous sound speed fields [13,14].

The development of data-driven sound speed modeling can be divided into three related directions. The first direction estimates or reconstructs sound speed profiles from limited acoustic, hydrographic, or remote sensing observations [15]. Early neural network inversion demonstrated that nonlinear mappings could be learned between surface variables and the subsurface sound speed structure [16]. Compressive sensing and EOF-based methods later reduced the dimensionality of the inversion problem and improved the profile reconstruction efficiency [17,18,19]. These methods are valuable when observations are sparse, but their performance depends strongly on predefined bases, measurement configurations, or profile-level representations.

The second direction focuses on temporal SSP prediction [20]. ConvLSTM, LSTM, CNN-BiLSTM-Attention, hierarchical LSTM, neural ordinary differential equations, and Transformer-based models have been introduced to capture historical dependence, nonlinear variation, and depth-specific behavior [21,22,23,24,25,26]. These studies show that recurrent and attention-based models can improve SSP prediction and that different depth layers may require different representations. Nevertheless, most of these methods predict one-dimensional profiles or long-term sequences at selected locations, rather than the complete next-day 3D field.

The third direction extends data-driven modeling to regional 3D reconstruction and spatiotemporal forecasting. Tensor neural networks and 3D convolutional models have been used to reconstruct full-depth fields while controlling model complexity and incorporating underwater vertical information [27,28]. ST-LSTM-SA, multi-spatial-scale deep learning, and STA-ConvLSTM-style methods further combine recurrent convolution and attention to model spatial coupling and temporal evolution [29,30,31]. These developments move the field from isolated SSP estimation toward regional field prediction. However, existing models are still generally optimized using profile-wide or field-wide errors, and the remaining errors in acoustically sensitive thermocline layers are rarely treated as a separate correction problem.

Beyond ocean acoustics, sensor-derived time-series forecasting provides a broader methodological context for the present study. Oyeleye et al. compared ARIMA, linear regression, support vector regression, *k*-nearest-neighbor regression, decision trees, random forest, and LSTM for heart rate prediction from wearable accelerometer time series, and they showed that classical statistical and machine learning models can remain competitive under sliding-window and walk-forward forecasting settings [32]. Staffini et al. directly compared autoregressive, LSTM, and ConvLSTM models on wearable heart rate sequences and found that the autoregressive model achieved the best performance across the examined settings [33]. Reiss et al. combined photoplethysmography and motion sensor signals with convolutional neural networks for large-scale heart rate estimation, highlighting the importance of robustness and generalization across sensor conditions and subjects [34]. Although physiological sensing and ocean sound speed prediction concern different physical systems, they share several methodological issues, including temporally ordered data, overlapping input windows, classical versus deep temporal baselines, and robustness to heterogeneous sensor-derived observations. This broader sensor forecasting perspective motivates the use of persistence, autoregressive, ARIMA, recurrent, convolutional, attention-based, and Transformer baselines in the present daily 3D forecasting study.

Despite these advances, two issues remain. Many deep learning models [35] are trained and evaluated mainly with full-field errors such as the overall RMSE or MAE, whereas localized errors in acoustically sensitive depth layers receive less attention. Sound speed errors are not vertically uniform. The thermocline usually has stronger vertical gradients and more active spatiotemporal variability, so local errors tend to concentrate there. For underwater sound propagation, changes in sound speed structure near the thermocline can alter acoustic ray refraction and propagation paths [1,36]. Global error metrics alone therefore do not fully describe model behavior in acoustically sensitive depth bands.

A second issue is the treatment of the full-depth 3D sound speed field as a single prediction target. Daily 3D fields contain a relatively stable background evolution, but thermocline-related residual errors are more depth-selective and gradient-related. A single end-to-end model may reduce the loss in the spatially dominant stable regions while leaving localized vertical errors under-corrected. A residual correction stage can address this imbalance, but its vertical guidance should adapt to the ocean state rather than rely on a manually prescribed correction interval. Studies on hierarchical LSTM and 3D reconstruction with underwater vertical information have shown that depth-dependent structure and vertical information are important for sound speed modeling [24,28]. The combination of global spatiotemporal prediction with prediction-time, full-depth, gradient-guided residual correction in daily 3D field prediction remains insufficiently studied.

To address these issues, this study proposes a daily 3D ocean sound speed prediction framework that combines a 3D ConvLSTM base predictor with the Full-Depth Dynamic Gradient-Guided Residual Correction Network (FDGRC-Net). The framework follows a two-stage modeling strategy. First, the 3D ConvLSTM base predictor learns the global spatiotemporal evolution from the preceding 60 daily 3D sound speed fields and generates a background prediction of the target-day sound speed field [25,37]. Then, a continuous full-depth mask is generated from the previous-day regional temperature gradient profile over all 40 retained physical depth levels. A six-channel residual feature stack is constructed using the base prediction, the previous observed field, the temporal increment, the vertical gradient of the base prediction, a depth embedding, and the dynamic mask. With the base predictor frozen, FDGRC-Net uses the mask for residual feature guidance, loss weighting, and output gating, thereby enhancing the correction of thermocline-related local errors without prescribing fixed upper and lower depth boundaries.

The contributions of this paper can be summarized as follows:

1. We identify a depth-localized error pattern in daily 3D ocean sound speed prediction. Depthwise diagnosis shows that the residual errors of the 3D ConvLSTM base predictor are mainly concentrated in thermocline-related layers and coincide with a strong temperature gradient structure, motivating targeted correction rather than uniform full-depth refinement.
2. We propose a two-stage framework for next-day 3D sound speed field prediction that separates global background forecasting from full-depth dynamic gradient-guided residual correction. The 3D ConvLSTM base predictor captures daily spatiotemporal evolution, while FDGRC-Net uses a prediction-time mask generated from the previous-day temperature profile to guide residual features, loss weighting, and output gating.
3. We demonstrate that dynamic gradient-guided residual correction improves the full-field prediction accuracy under the daily 3D prediction setting. Mechanism ablations distinguish the effects of residual learning and gradient guidance. Compared with ConvLSTM-60d, FDGRC-Net reduces the overall RMSE from 0.4209 m/s to 0.3916 m/s and the overall MAE from 0.2302 m/s to 0.2180 m/s. Four matched-seed experiments further assess initialization robustness.
4. We evaluate the framework in the northern South China Sea, over six frozen out-of-time years and ONI-defined ENSO states, against 521 collocated Argo profiles, and through a controlled Bellhop propagation diagnostic. These experiments examine regional, temporal, climate-state, observation-based, and acoustic consistency without changing the core full-depth formulation.

The remainder of this paper is organized as follows. Section 2 introduces the study areas, data sources, sound speed calculation method, data preprocessing procedure, compared methods, the proposed 3D ConvLSTM + FDGRC-Net method, and the extended evaluation protocols. Section 3 presents the experimental results, including the selection of the base predictor, comparison with representative and classical baselines, mechanism ablations, multi-seed analysis, cross-region and out-of-time evaluations, Argo consistency assessment, Bellhop diagnostic, and computational complexity. Section 4 discusses the residual correction mechanism, dynamic gradient guidance, model robustness, observation-based and acoustic interpretation, comparison with existing sound speed prediction studies, and model limitations. Section 5 concludes the paper and outlines future research directions.

## 2. Materials and Methods

### 2.1. Data

**Figure 1:**
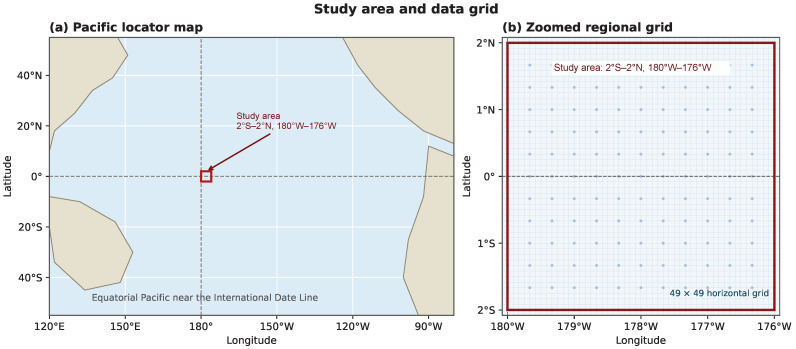
Location of the study area and data grid. (**a**) Pacific locator map showing the selected equatorial Pacific region near the International Date Line. (**b**) Zoomed regional grid covering 2° S–2° N and 180° W–176° W on a 49×49 horizontal grid.

This study uses daily-mean 3D ocean temperature and salinity data to construct samples for ocean sound speed field prediction. The study area is a low-latitude equatorial Pacific region near the International Date Line [38,39], bounded by 2° S–2° N and 180° W–176° W (Figure 1). The source is the Copernicus Marine Global Ocean Physics Reanalysis, product ID GLOBAL_MULTIYEAR_PHY_001_030, version GLORYS12V1. This Level-4 product provides daily and monthly fields on a regular 1/12° grid with 50 standard depth levels [13]. The present study uses the daily sea-water potential temperature (thetao) and salinity (so) fields from 1 January 2000 to 28 December 2009.

The regional subset was extracted directly from the native signed-coordinate grid using −2°≤ϕ≤2° and −180°≤λ≤−176°. Boolean indexing produced 49 latitude points and 49 longitude points. No longitude wrapping, dateline stitching, regridding, nearest-neighbor interpolation, or linear spatial interpolation was applied.

The first 40 native GLORYS12 depth levels were retained, extending from 0.4940 m to 1941.8929 m. Their values are listed in Table 1.

As shown in Table 1, the vertical spacing increases with depth and is therefore non-uniform. The native depth coordinates are retained throughout the experiments, without vertical regridding.

Based on the temperature, salinity, and depth information in the study region, the UNESCO sound speed equation is used to calculate the sound speed at each valid grid point [11,12]. The resulting daily sound speed fields are then organized as a continuous 3D sound speed field sequence. The study region is represented on a regular 3D grid, and each time step corresponds to one 3D sound speed field. In this study, the spatial size of each 3D sound speed field is 40×49×49, where 40 denotes the number of vertical depth levels and 49×49 denotes the horizontal grid size. Therefore, the sound speed field at day *t* can be expressed as

(1) $$C_{t}\in R^{D\times H\times W},$$

where *D*, *H*, and *W* denote the numbers of depth, latitudinal, and longitudinal grid points, respectively. In the present experiments, D=40, H=49, and W=49.

The prediction task is defined as follows: given a historical sequence of *L* consecutive daily 3D sound speed fields before the target time, the model predicts the 3D sound speed field at the target day *t*. The historical input sequence can be written as

(2) $$\{C_{t-L},C_{t-L+1},\ldots ,C_{t-1}\},$$

where *L* is the historical window length. According to the window-length sensitivity experiment, the final model uses L=60, i.e., the past 60 daily 3D sound speed fields are used to predict the next-day 3D sound speed field.

To allow the model to distinguish valid ocean grid points from invalid grid points, a valid mask is also introduced at the input side. Each historical time step contains two channels: the normalized sound speed field and the corresponding valid mask. Therefore, the input tensor of the 3D ConvLSTM base predictor is organized as

(3) B×60×2×40×49×49,

where *B* is the batch size, 60 is the number of historical time steps, 2 is the number of input channels, and 40×49×49 is the 3D spatial grid size.

### 2.2. Dataset Preprocessing

The seawater sound speed is calculated at each valid grid point using the UNESCO-style equation [11,12]:

(4) $$C_{t}\left(z,h,w\right)=f_{\mathrm{UNESCO}}\left(T_{t}\left(z,h,w\right),S_{t}\left(z,h,w\right),p\left(z\right)\right).$$

Here, $T_{t}$ is the potential temperature, $S_{t}$ is the salinity, and the pressure term is approximated from the native geometric depth *z* in meters [40] as

(5) p(z)=0.1z,

with *p* expressed in bar. No latitude-dependent pressure conversion or TEOS-10 transformation is applied. The converted fields are stored in m/s.

A valid mask $V_{t}$ is then constructed for each 3D sound speed field to avoid the influence of land areas or missing values during model training and evaluation. The mask value is set to 1 for valid ocean grid points and 0 for land or missing grid points. All 49×49=2401 surface cells are valid. Of the 2401 horizontal columns, 2397 remain valid at all 40 depths; the remaining four contain bottom invalid cells. Specifically, two cells are invalid at 1684.2841 m and four cells are invalid at 1941.8929 m. The valid mask is used as a model input and in all loss functions and evaluation metrics, so these bottom invalid cells do not contribute to optimization or reported errors.

The sound speed fields are normalized to improve the training stability. In the implementation, standardization based on training-set statistics is used:

(6) $$C_{t}^{\mathrm{norm}}=\frac{C_{t}-\mu_{\mathrm{train}}}{\sigma_{\mathrm{train}}},$$

where $\mu_{\mathrm{train}}$ and $\sigma_{\mathrm{train}}$ are the mean and standard deviation calculated from the training set. The same normalization parameters are used for the validation and test sets to avoid information leakage from future data. After prediction, the model outputs are transformed back to physical units for evaluation.

Supervised learning samples are generated using a sliding-window strategy. For a target day *t*, the model input consists of the normalized sound speed fields and valid masks from the previous 60 days, and the label is the normalized sound speed field at day *t*:

(7) $$X_{t}=\left\{\left(C_{t-60}^{\mathrm{norm}},V_{t-60}\right),\ldots ,\left(C_{t-1}^{\mathrm{norm}},V_{t-1}\right)\right\},$$

(8) $$Y_{t}=C_{t}^{\mathrm{norm}}.$$

Sliding-window sampling is commonly used in sequence prediction tasks to transform continuous time series into supervised input–target pairs [30,37].

Finally, the dataset is divided chronologically into training, validation, and test sets. Random splitting is not used because it would mix earlier and later ocean states and could introduce future information into model development. The daily fields are sorted by date and assigned zero-based indices from 0 to 3649, where index 0 corresponds to 1 January 2000 and index 3649 corresponds to 28 December 2009. To ensure that all window-length experiments, including the 365-day configuration, use identical target dates, the first prediction target is fixed at index 365. This leaves 3285 target samples, divided chronologically into 2628 training, 328 validation, and 329 test targets, corresponding approximately to an 80:10:10 split.

A date continuity audit found no missing dates, duplicate dates, or uneven temporal intervals; all adjacent fields are separated by one day, and no temporal interpolation or missing-day filling was required. Table 2 gives the exact zero-based target indices, calendar dates, sample counts, and roles of each subset.

Each target uses the immediately preceding 60 daily fields. The first training sample uses input indices 305–364, corresponding to 1 November–30 December 2000, to predict target index 365 on 31 December 2000. The first validation sample uses input indices 2933–2992, corresponding to 12 January–11 March 2008, to predict target index 2993 on 12 March 2008. The first test sample uses input indices 3261–3320, corresponding to 5 December 2008–2 February 2009, to predict target index 3321 on 3 February 2009. Adjacent samples share 59 historical fields but have distinct targets. Subset assignment is determined by the target date; preceding observations may provide the input history at a boundary, but validation and test targets are never used to update model parameters. The training loader shuffles samples within each epoch, whereas the validation and test loaders preserve the chronological order.

### 2.3. Overall Framework of the Proposed Method

Given the preceding 60 daily sound speed fields, the base predictor produces

(9) $${\hat{C}}_{t}^{\mathrm{base}}=F_{\mathrm{base}}\left(X_{t}\right).$$

The residual corrector receives a depth-aware feature stack $Z_{t}$ and predicts a residual field

(10) $$R_{t}=F_{\mathrm{res}}\left(Z_{t}\right).$$

**Figure 2:**
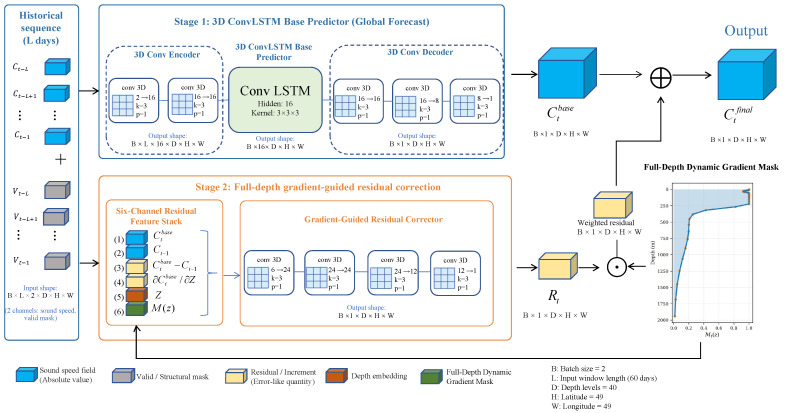
Architecture of FDGRC-Net. Stage 1 uses the preceding 60 sound speed fields and validity masks to produce a next-day background prediction with a 3D ConvLSTM. Stage 2 forms a six-channel residual feature stack and predicts a residual field. The full-depth dynamic mask $M_{t}\left(z\right)$, generated from the previous-day temperature gradient profile, enters the feature stack and gates the residual before addition to the frozen base prediction.

The final prediction is

(11) $${\hat{C}}_{t}^{\mathrm{final}}={\hat{C}}_{t}^{\mathrm{base}}+M_{t}\left(z\right)⊙R_{t},$$

where $M_{t}\left(z\right)$ is the prediction-time mask generated from the previous-day temperature profile and broadcast across the horizontal grid. Figure 2 shows the proposed framework.

### 2.4. 3D ConvLSTM Base Predictor

The 3D ConvLSTM base predictor is used to capture spatial structures and temporal dependencies in historical 3D sound speed field sequences. Differing from conventional LSTM, ConvLSTM replaces fully connected operations with convolutional operations, allowing the model to retain the spatial structure while modeling temporal evolution [37,41]. Since this study deals with 3D sound speed fields, 3D convolutions and a 3D ConvLSTM structure are adopted to jointly model local correlations along depth, latitude, and longitude. ConvLSTM-based models have also been shown to be effective for ocean sound speed prediction tasks [25,30].

The base predictor consists of three main parts: a 3D convolutional encoder, a 3D ConvLSTM temporal modeling module, and a 3D convolutional decoder. First, the 3D convolutional encoder extracts spatial features from the input field at each historical time step. The input channels include the normalized sound speed field and the valid mask. The encoder maps the input into a higher-dimensional feature space through consecutive 3D convolutional layers. The encoder contains two 3D convolutional layers that map the input from two channels to 16 channels and then maintain 16 channels. The kernel size is 3×3×3, and group normalization and GELU activation are used after convolution to improve the training stability and nonlinear representation capabilities.

The retained GLORYS12 depth levels are non-uniformly spaced. Accordingly, the 3×3×3 convolutions operate over neighboring depth indices rather than over a constant physical thickness in meters. This preserves the native vertical ordering and local adjacency of the depth levels, while the physical vertical span of the convolutional receptive field varies with depth. The native grid is retained to avoid introducing an additional vertical interpolation step.

The encoded historical feature sequence is then passed to the 3D ConvLSTM module. This module computes the input gate, forget gate, output gate, and candidate state using 3D convolutional operations and recursively updates the hidden state along the temporal dimension. For a time step *t*, the update process of the 3D ConvLSTM can be written as

(12a) $$\begin{array}{cc} i_{t} & =\sigma (W_{xi}*X_{t}+W_{hi}*H_{t-1}+b_{i}), \end{array}$$

(12b) $$\begin{array}{cc} f_{t} & =\sigma (W_{xf}*X_{t}+W_{hf}*H_{t-1}+b_{f}), \end{array}$$

(12c) $$\begin{array}{cc} {\tilde{Q}}_{t} & =\tanh(W_{xc}*X_{t}+W_{hc}*H_{t-1}+b_{c}), \end{array}$$

(12d) $$\begin{array}{cc} Q_{t} & =f_{t}⊙Q_{t-1}+i_{t}⊙{\tilde{Q}}_{t}, \end{array}$$

(12e) $$\begin{array}{cc} o_{t} & =\sigma (W_{xo}*X_{t}+W_{ho}*H_{t-1}+b_{o}), \end{array}$$

(12f) $$\begin{array}{cc} H_{t} & =o_{t}⊙\tanh\left(Q_{t}\right), \end{array}$$

where ∗ denotes 3D convolution; ⊙ denotes element-wise multiplication; $i_{t}$, $f_{t}$, and $o_{t}$ are the input, forget, and output gates, respectively; and $Q_{t}$ and $H_{t}$ denote the ConvLSTM cell state and hidden state. The symbol $Q_{t}$ is used for the recurrent cell state to distinguish it from the sound speed field $C_{t}$. In this study, a one-layer 3D ConvLSTM is used, with 16 hidden channels and a convolutional kernel size of 3×3×3.

Finally, the 3D convolutional decoder maps the hidden features output by the 3D ConvLSTM back to a single-channel 3D sound speed field. The decoder contains three 3D convolutional layers that map the hidden representation from 16 channels to 16 channels, then to 8 channels, and finally to one output channel. The first two decoder layers use group normalization and GELU activation, while the last layer uses a linear output to generate the base prediction ${\hat{C}}_{t}^{\mathrm{base}}$.

### 2.5. Full-Depth Gradient-Guided Residual Corrector

The residual feature stack contains the base prediction, the previous observed sound speed field, the temporal increment, the physical vertical gradient of the base prediction, the normalized physical depth, and the dynamic depth mask:

(13) $$Z_{t}=\left[{\hat{C}}_{t}^{\mathrm{base}},\;C_{t-1},\;{\hat{C}}_{t}^{\mathrm{base}}-C_{t-1},\;\frac{\partial {\hat{C}}_{t}^{\mathrm{base}}}{\partial z},\;z_{\mathrm{norm}},\;M_{t}\left(z\right)\right].$$

The depth embedding is constructed from the native physical depths, and the vertical gradient channel uses the corresponding non-uniform depth intervals. The residual corrector consists of four 3D convolutional layers with channel dimensions 6→24→24→12→1. During second-stage training, the base ConvLSTM parameters remain frozen and only the residual branch is optimized.

### 2.6. Full-Depth Dynamic Gradient Mask Generator

For each target day *t*, the previous-day temperature field is horizontally averaged over valid regional grid points:

(14) $${\bar{T}}_{t-1}\left(z_{k}\right)=\frac{\sum_{h,w}V_{t-1}\left(z_{k},h,w\right)T_{t-1}\left(z_{k},h,w\right)}{\sum_{h,w}V_{t-1}\left(z_{k},h,w\right)}.$$

On the non-uniform depth grid, adjacent interval gradients are calculated as

(15) $$g_{t,k}=\left|\frac{{\bar{T}}_{t-1}\left(z_{k+1}\right)-{\bar{T}}_{t-1}\left(z_{k}\right)}{z_{k+1}-z_{k}}\right|.$$

They are mapped back to the 40 depth levels using nearest-interval values at the endpoints and the mean of adjacent interval gradients at interior levels:

(16) $$G_{t,0}=g_{t,0},\;\;G_{t,D-1}=g_{t,D-2},\;\;G_{t,k}=\frac{g_{t,k-1}+g_{t,k}}{2}.$$

The resulting profile is smoothed with a one-dimensional Gaussian-like kernel with σ=1 depth level. It is then normalized over the complete retained water column:

(17) $$G_{t}^{\mathrm{norm}}\left(z\right)=\frac{G_{t}^{\mathrm{smooth}}\left(z\right)}{\max_{z}G_{t}^{\mathrm{smooth}}\left(z\right)+\epsilon }.$$

The continuous dynamic mask is

(18) $$M_{t}\left(z\right)={\left[\mathrm{clip}\left(\frac{G_{t}^{\mathrm{norm}}\left(z\right)}{\tau },0,1\right)\right]}^{\gamma },$$

where τ=0.4, γ=1.0, and $\epsilon =10^{-8}$. A gradient intensity of at least 40% of the daily full-depth maximum therefore receives unit weight, whereas weaker gradient levels receive continuous weights between zero and one.

**Figure 3:**
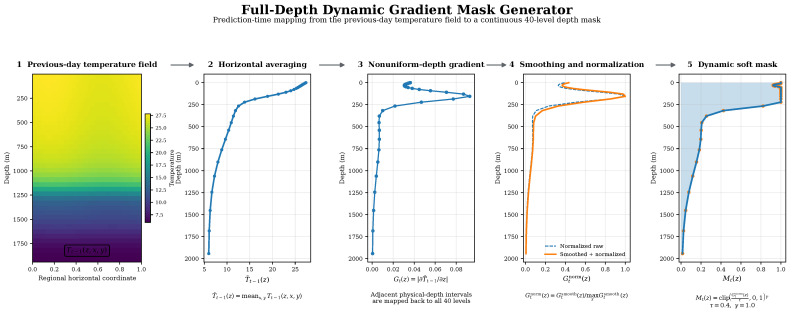
Generation of the full-depth dynamic gradient mask. The previous-day temperature field is horizontally averaged to obtain a regional profile, differentiated on the non-uniform physical depth grid, smoothed along the depth, normalized by the maximum gradient over all 40 retained levels, and transformed into a continuous soft mask. The illustrated curves are schematic; the actual mask is calculated separately for each target date.

The complete mask-generation process is illustrated in Figure 3.

### 2.7. Full-Depth Climatological Mask Ablation

To separate the effect of gradient-guided localization from that of day-specific adaptation, a climatological variant is retained as a principal ablation. It uses the mean regional temperature profile over the development period (training and validation target dates) and applies the same gradient, smoothing, normalization, and soft-mapping operations. The resulting fixed 40-level mask is shared by all samples. Test targets and test errors are not used in its construction.

### 2.8. Full-Depth No-Mask Residual Ablation

A no-mask residual model is used to separate the effect of adding a residual branch from the effect of gradient-guided localization. It retains the same residual corrector architecture, but the sixth feature channel is set to one, and the predicted residual is added over the full water column without output gating,

(19) $${\hat{C}}_{t}^{\text{no-mask}}={\hat{C}}_{t}^{\mathrm{base}}+R_{t},$$

and no fixed depth interval or gradient-derived mask is used. This ablation is trained with the masked full-field loss only and is selected by its validation full-field loss.

### 2.9. Training Strategy and Loss Function

Experiments used Python 3.10.13, PyTorch 2.1.1+cu118, CUDA 11.8, and cuDNN 8.7.0.The ConvLSTM base predictor is first optimized using the masked full-field mean squared error. The selected base checkpoint is then frozen for all second-stage experiments. For the two gradient-guided residual variants, the objective is

(20) $$L_{\mathrm{guided}}=L_{\mathrm{all}}+\lambda_{M}L_{M},$$

where

(21) $$L_{\mathrm{all}}=\frac{\sum V_{t}⊙{\left({\hat{C}}_{t}^{\mathrm{final}}-C_{t}\right)}^{2}}{\sum V_{t}},$$

and

(22) $$L_{M}=\frac{\sum V_{t}⊙M_{t}\left(z\right)⊙{\left({\hat{C}}_{t}^{\mathrm{final}}-C_{t}\right)}^{2}}{\sum V_{t}⊙M_{t}\left(z\right)+\epsilon }.$$

For FDGRC-Net, $M_{t}\left(z\right)$ is generated from $T_{t-1}$ for each sample; for the climatological ablation, the fixed development-period mask is used. Both guided variants use $\lambda_{M}=1.0$, whereas the no-mask residual ablation uses $L_{\mathrm{all}}$ alone.

All residual variants use AdamW [42] with a learning rate of $10^{-4}$, weight decay of $10^{-5}$, batch size 2, automatic mixed precision, gradient clipping at 1.0, and a maximum of 30 epochs. The best checkpoint for each variant is selected from the validation set using the same loss definition employed during its training.

### 2.10. Evaluation Indicators

The principal field accuracy measures are the overall RMSE and overall MAE in physical units. Vertical behavior is evaluated using the pooled depthwise RMSE, upper 300 m RMSE (z≤300 m), below 300 m RMSE (z>300 m), and peak depth RMSE within the upper 300 m. To define this regional evaluation partition from physical data, we analyzed the daily regional mean temperature gradient profiles over the 2956-day development period using the same full-depth, non-uniform depth gradient operator employed by FDGRC-Net. The thermocline gradient peak had a median depth of 155.8507 m and a 95th-percentile depth of 186.1256 m, and all 2956 daily peaks occurred within the upper 300 m. With the normalized gradient threshold set to 0.4, the deepest active gradient depth had a median of 222.4752 m and a 95th percentile of 266.0403 m; all active gradient depths remained within 300 m on 99.7632% of development days. The development-period mean gradient peaked at 155.8507 m, and its normalized values of at least 0.4 extended from 109.7000 to 222.4752 m. These statistics show that the thermocline gradient peaks and principal high-gradient depths were concentrated within the upper 300 m; therefore, 300 m was adopted as the post hoc regional diagnostic partition. The peak depth RMSE measures the error in the depth of the strongest absolute vertical sound speed gradient within the upper 300 m, using the same non-uniform depth operator for prediction and reference fields. The full depthwise RMSE profile is additionally reported to show where errors are reduced.

For the Argo evaluation, each collocated profile is assessed using the overall RMSE, overall MAE, upper 300 m RMSE, mean bias, and profile correlation. GLORYS12 and all model fields are interpolated to the valid Argo observation depths, so errors are calculated only where observations are available. For the acoustic diagnostic, the principal measures are the daily transmission loss (TL) MAE, median absolute TL error, 90th-percentile absolute TL error, energy-weighted mean arrival time error, and RMS delay spread error. Lower values indicate closer agreement with the corresponding observational or Bellhop reference.

### 2.11. Statistical Uncertainty and Paired Consistency Analysis

Random-seed robustness is summarized using the mean and standard deviation across the four completed matched-seed runs. We additionally report the paired ordering relative to the corresponding same-seed ConvLSTM checkpoint to assess whether the observed improvements remain stable across initializations.

For the frozen 2010–2015 and acoustic evaluations, each target day is treated as the paired statistical unit. Let $r_{m}\left(t\right)$ denote the daily error of model *m* under the metric of interest. The paired reduction relative to ConvLSTM-60d is

(23) $$d_{m}\left(t\right)=r_{\mathrm{ConvLSTM}}\left(t\right)-r_{m}\left(t\right),$$

where positive values indicate improvement. The improved-day fraction is the proportion of dates for which $d_{m}\left(t\right)>0$. To account for short-range temporal dependence, 95% confidence intervals for the mean daily reduction are estimated from 10,000 paired circular moving-block bootstrap replicates with a 7-day block length. The same resampled date indices are applied to both models. A one-sided Wilcoxon signed-rank test is retained as a secondary paired consistency check; interpretation in the manuscript is based primarily on the block bootstrap interval. Annual and ENSO analyses are performed without retraining or test-driven parameter adjustment.

For the Argo evaluation, the paired statistical unit is the WMO float rather than an individual depth sample. A 95% confidence interval is estimated using 5000 WMO float-level cluster bootstrap replicates with seed 20260720; all profiles from a resampled float are retained together. Positive paired differences indicate lower profile errors than for ConvLSTM-60d.

### 2.12. Compared Methods

To evaluate the proposed method under a controlled setting, we compare it with standard persistence, anomaly persistence, EOF-AR, EOF-ARIMA, ConvLSTM-60d, CNN-BiLSTM-style, H-LSTM-style, STA-ConvLSTM-like, TimeSformer3D-60d [43], and 3D U-Net-60d [44] baselines. The first four methods provide non-learning or classical statistical references, whereas the remaining learned baselines represent recurrent, attention-based, convolutional, and video Transformer forecasting strategies. Several literature-related neural models were originally designed for SSP prediction, SSP inversion, few-shot profile prediction, or monthly multi-spatial-scale forecasting, rather than for daily 3D sound speed field prediction. Therefore, the exact reproduction of their original experimental settings is not suitable for the present task. Table 3 summarizes how these methods were implemented or adapted to the common daily 3D forecasting setting.

The classical baselines are implemented directly under the same GLORYS12-derived daily 3D sound speed data, chronological split, and next-day evaluation protocol. The literature-related learned methods are implemented as task-adapted baselines: their main architectural ideas are retained, while the input and output interfaces are modified to use the same 60-day input history, next-day 3D prediction target, normalization strategy, and evaluation metrics. These learned baselines use the same input tensor size described above, with two channels denoting the normalized sound speed field and the valid data mask, and output a next-day 3D field with size

(24) B×1×40×49×49.

Therefore, the comparisons in this study should be understood as comparisons among representative modeling strategies under the same daily 3D sound speed field prediction setting, rather than as exact reproductions of the numerical results reported in the original papers.

### 2.13. Cross-Region Evaluation Protocol

A second regional experiment is conducted in the northern South China Sea, bounded by 18–22° N and 116–120° E. It uses the same GLORYS12-derived daily field construction, 40 retained depth levels, 60-day input window, chronological target split, network architectures, optimization settings, mask formula, and evaluation code as the equatorial Pacific experiment. The models are retrained on the South China Sea data; this experiment therefore evaluates architecture- and mechanism-level transferability rather than zero-shot parameter transfer. The mask parameters τ, γ and the smoothing width are unchanged.

### 2.14. Out-of-Time and ENSO Evaluation Protocol

Temporal robustness is evaluated on a clean daily extension from 1 January 2010 to 31 December 2015. The ConvLSTM-60d, full-depth climatological, and full-depth dynamic checkpoints developed from the 2000–2009 period are frozen throughout this evaluation. The 2010–2015 fields are used only as historical inputs and prediction targets; they are not used for training, checkpoint selection, normalization, hyperparameter selection, or climatological mask updating. The climatological mask remains fixed from the training-plus-validation development period, whereas the dynamic mask is generated from the previous-day temperature profile for each target date.

ENSO-state robustness [45] is assessed using the NOAA Climate Prediction Center Oceanic Niño Index (ONI), a three-month running mean of Niño-3.4 sea surface temperature anomalies [46]. Each target date is assigned the ONI value centered on its calendar month. Dates with ONI ≥ 0.5 °C are categorized as El Niño conditions, dates with ONI ≤ −0.5 °C as La Niña conditions, and the remainder as neutral conditions. This yields 547 El Niño, 609 La Niña, and 1035 neutral target days. The labels are used only for stratified evaluation.

### 2.15. Argo Observation-Based Consistency Protocol

Observation-based consistency is evaluated using quality-controlled Argo profiles from 1 January 2010 to 31 December 2015 within the equatorial Pacific model domain [14]. The official Argo GDAC search matched 531 files. Decoding produced 728 inventory profiles, of which 564 passed the prescribed temperature, salinity, pressure, location, and vertical coverage checks. After date matching and spatial collocation with the frozen prediction fields, 521 profiles from 67 WMO floats and 461 unique prediction dates remained. No download or decoding failures occurred.

Adjusted pressure, temperature, and salinity variables are used when available, with quality control flags restricted to accepted values. The Argo-derived sound speed is calculated using the same UNESCO 1983 implementation used to construct the GLORYS12 target fields. The primary analysis uses the bilinear horizontal interpolation of GLORYS12 and the three frozen model outputs to each Argo location, followed by vertical interpolation to the valid observed depths. No observational values are extrapolated or filled. A nearest-grid evaluation is retained as a collocation sensitivity analysis, yielding 2084 method–profile rows. Argo data are not used for training, normalization, checkpoint selection, or hyperparameter adjustment. Because Argo observations may contribute to the GLORYS12 assimilation system, this experiment is described as an observation-based consistency evaluation rather than a fully independent benchmark.

### 2.16. Controlled Acoustic Propagation Protocol

A controlled range-independent Bellhop experiment is used to examine whether reduced sound speed errors produce measurable propagation-level differences [1]. The acoustic simulations used a locally built BELLHOP executable from the Acoustics Toolbox (available online: https://oalib-acoustics.org/website_resources/AcousticsToolbox/, accessed on 30 July 2026). The evaluation includes all 329 formal 2009 test dates. For each date, the regional center-point sound speed profiles from the GLORYS12-derived reference field, ConvLSTM-60d, the full-depth climatological variant, and FDGRC-Net are propagated under identical acoustic and boundary settings. No model is retrained or selected using Bellhop output.

Bellhop is run at 500 Hz with a source depth of 100 m. TL is evaluated over receiver ranges of 5–50 km at 1 km spacing and receiver depths of 0–500 m at 10 m spacing using incoherent Gaussian beam output. TL values are clipped at 120 dB before error calculation; 100 and 140 dB clipping levels are used as sensitivity checks. Arrival diagnostics are evaluated at ranges of 10, 20, 30, 40, and 50 km and depths of 50, 100, 150, 200, 300, and 500 m. For valid arrivals with complex amplitude $a_{k}$ and travel time $t_{k}$, the energy-weighted mean arrival time is

(25) $$\bar{t}=\frac{\sum_{k}{\left|a_{k}\right|}^{2}t_{k}}{\sum_{k}{\left|a_{k}\right|}^{2}},$$

and the RMS delay spread is

(26) $$\tau_{\mathrm{rms}}=\sqrt{\frac{\sum_{k}{\left|a_{k}\right|}^{2}{\left(t_{k}-\bar{t}\right)}^{2}}{\sum_{k}{\left|a_{k}\right|}^{2}}}.$$

A receiver contributes to the paired arrival analysis only when valid arrivals are available for both the predicted and reference environments. Because all non-SSP inputs are held fixed, the experiment isolates sensitivity to the predicted vertical sound speed structure. It is interpreted as a controlled diagnostic rather than as field-calibrated operational validation.

## 3. Results

Unless otherwise stated, equatorial Pacific results are reported on the 329-date held-out test set after all model development and checkpoint selection were completed using only the training and validation subsets in Table 2.

### 3.1. Experimental Configuration and Selection of the Base Predictor

**Figure 4:**
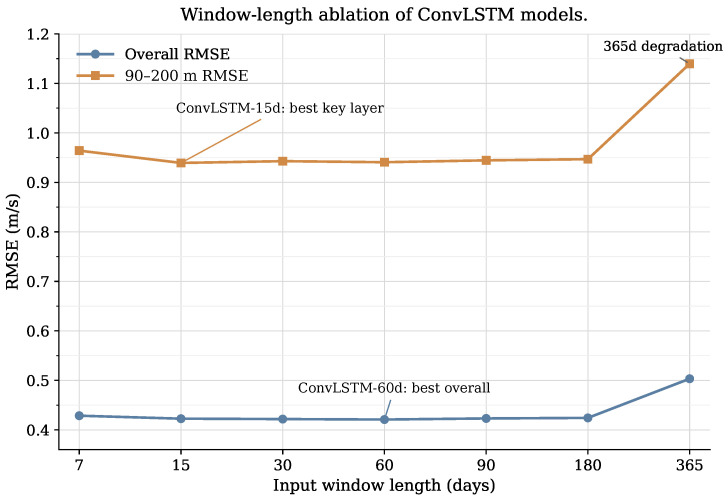
Window length ablation of ConvLSTM models. The 60-day input window achieves the lowest overall RMSE, whereas the 365-day window shows clear performance degradation.

The first experiment was conducted to determine a suitable historical input length for the 3D ConvLSTM base predictor. Table 4 and Figure 4 compare seven input window lengths, namely 7, 15, 30, 60, 90, 180, and 365 days. The ConvLSTM-60d model achieves the lowest overall RMSE of 0.4209 m/s, indicating that a 60-day historical sequence provides the most stable full-field prediction among the tested settings.

This distinction is important because FDGRC-Net is designed as a residual correction framework built upon a stable background prediction. The base predictor should therefore provide the most reliable full-field estimate before full-depth gradient-guided residual correction is applied. The 365-day window performs substantially worse, with the overall RMSE increasing to 0.5033 m/s. This result suggests that simply increasing the historical window length does not necessarily improve next-day prediction and may introduce redundant low-frequency information or optimization difficulty. Based on these results, ConvLSTM-60d is selected as the frozen base predictor for all subsequent FDGRC-Net experiments.

### 3.2. Thermocline Statistics and the 300 m Diagnostic Partition

**Figure 5:**
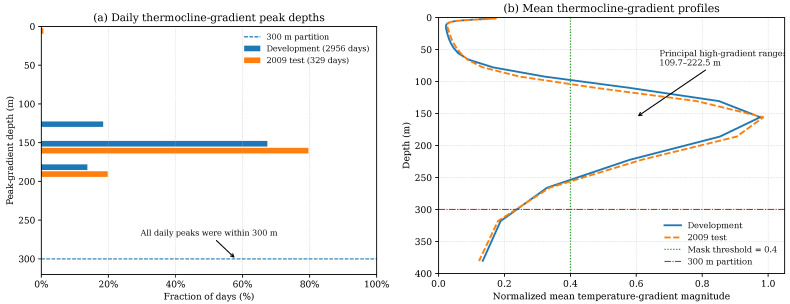
Development-period and test-period thermocline gradient statistics supporting the regional 300 m diagnostic partition. (**a**) Distribution of daily peak gradient depths. (**b**) Development-period and test-period mean normalized temperature gradient profiles. The 300 m reference is used as a regional evaluation partition rather than as a universal thermocline boundary.

The development-period temperature gradient statistics provide an independent physical basis for the regional evaluation partition. Across 2956 development days, all daily gradient peaks occur within the upper 300 m, with a median depth of 155.8507 m and a 95th-percentile depth of 186.1256 m. At the 0.4 activity threshold, all active gradient depths remain within 300 m on 99.7632% of development days. The 329-day test period shows the same peak depth distribution and a mean normalized high-gradient range of approximately 109.7–222.5 m. Figure 5 therefore supports using 300 m as an equatorial Pacific diagnostic partition rather than as a universal thermocline boundary.

### 3.3. Depthwise Error Diagnosis of the Base Predictor

**Figure 6:**
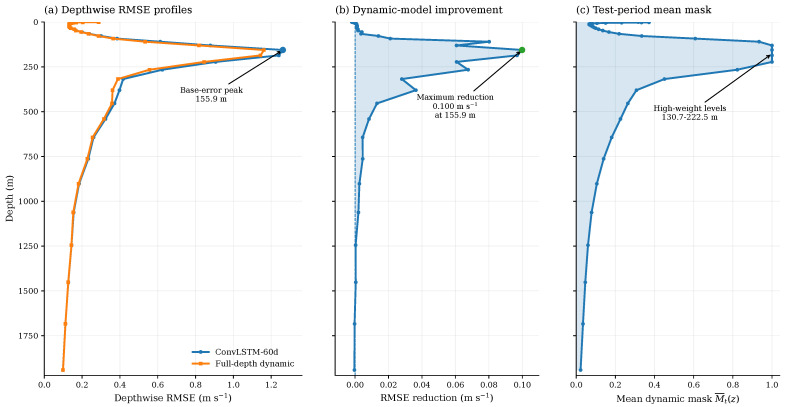
Depthwise error diagnosis of the ConvLSTM-60d base predictor and the full-depth dynamic gradient-guided model. (**a**) Depthwise RMSE profiles over the 40 retained physical depth levels. (**b**) RMSE reduction relative to ConvLSTM-60d, where positive values indicate improved prediction accuracy. (**c**) Test-period mean dynamic mask. The comparison shows that the largest base model error and the largest correction occur near the same thermocline-related depth, while the mask remains a continuous full-depth weighting profile.

The second experiment diagnoses where the ConvLSTM-60d base predictor still fails after achieving competitive full-field accuracy. As shown in Figure 6, the depthwise RMSE profile is strongly non-uniform over depth. The largest error occurs near 155.8507 m, and the neighboring thermocline-related levels also exhibit elevated errors. This indicates that the base predictor does not fail uniformly over the full water column; instead, its main residual errors are localized around thermocline-related layers.

Figure 6 further compares the base predictor with the final full-depth dynamic model and separately displays the resulting depthwise RMSE reduction and the test-period mean dynamic mask. The largest reduction also occurs near 155.8507 m, where the base model exhibits its maximum error. The mean mask assigns its highest weights mainly to approximately 130.7–222.5 m, while remaining a continuous full-depth profile rather than a prescribed interval. The 329-day test-period temperature gradient statistics confirm the same vertical regime: all daily gradient peaks occurred within the upper 300 m; the median and 95th-percentile peak depths were 155.8507 and 186.1256 m, respectively; and all active gradient depths remained within 300 m on 100% of test days at the 0.4 threshold. This correspondence supports the interpretation that the temperature gradient mask localizes the residual correction toward dynamically active layers. The mask itself is generated independently from the previous-day temperature profile and is not constructed from the test error curve.

### 3.4. Comparison with Representative Baselines

**Figure 7:**
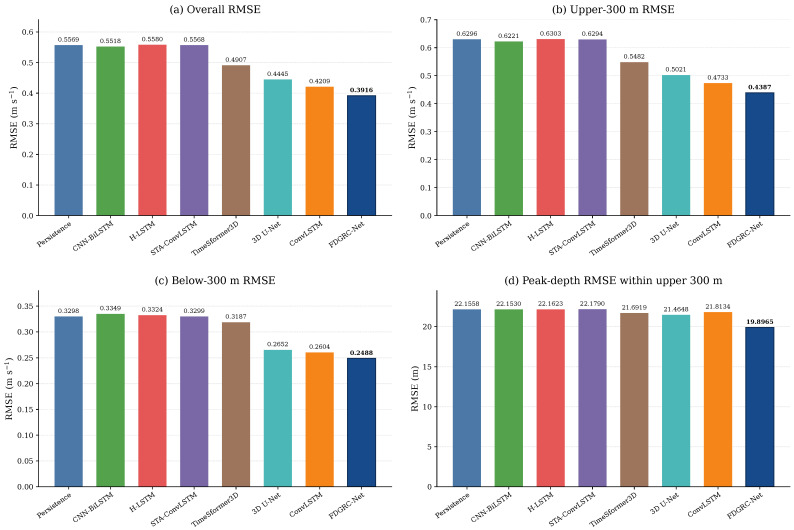
Comparison of the principal equatorial Pacific test metrics: (**a**) overall RMSE, (**b**) upper 300 m RMSE, (**c**) below 300 m RMSE, and (**d**) peak depth RMSE within the upper 300 m. Lower values indicate better performance. Colors distinguish the eight methods and are used consistently across all panels; bold numerical labels indicate the lowest (best) value in each panel.

Table 5 reports the full numerical comparison under the same daily 3D sound speed prediction setting, while Figure 7 visualizes the principal evaluation metrics. Because several reference architectures were originally developed for SSP-level or different-dimensional prediction tasks, we implemented task-adapted baselines following their main architectural ideas for fair comparison in the present 3D field prediction problem.

The improvement from Persistence to ConvLSTM-60d shows the value of spatiotemporal deep learning for daily sound speed forecasting. The overall RMSE decreases from 0.5569 to 0.4209 m/s, indicating that the standalone ConvLSTM-60d baseline captures much of the background temporal evolution. The CNN-BiLSTM-style, H-LSTM-style, STA-ConvLSTM-like, TimeSformer3D-60d, and 3D U-Net-60d baselines do not outperform ConvLSTM-60d in this task. TimeSformer3D-60d obtains an overall RMSE of 0.4907 m/s. This result supports the use of recurrent convolutional modeling as the base predictor for the present daily 3D forecasting task. FDGRC-Net adds a further improvement on top of the already strong ConvLSTM-60d base predictor.

Compared with ConvLSTM-60d, the proposed FDGRC-Net reduces the overall RMSE from 0.4209 to 0.3916 m/s and the overall MAE from 0.2302 to 0.2180 m/s. The upper 300 m RMSE decreases from 0.4733 to 0.4387 m/s, corresponding to a reduction of 7.3104%, while the below 300 m RMSE decreases from 0.2604 to 0.2488 m/s, a reduction of 4.4547%. The peak depth RMSE is also reduced from 21.8134 to 19.8965 m. The simultaneous improvement in the full-field, upper ocean, deeper layer, and peak depth metrics indicates that FDGRC-Net does not trade global stability for localized accuracy; instead, it improves thermocline-related prediction while preserving the overall background field.

### 3.5. Additional Classical Statistical Baselines

To complement the neural and task-adapted comparisons, we further evaluated three classical forecasting variants under the same chronological test protocol: anomaly persistence, EOF-AR, and EOF-ARIMA. This comparison is also consistent with broader sensor time-series studies showing that autoregressive and other classical models can remain competitive with deep recurrent architectures [32,33]. Standard persistence directly carries the most recent field forward, whereas anomaly persistence carries forward the latest departure from the training-period climatological background. The two EOF-based methods first project the 3D sound speed fields onto a training-only EOF basis and then forecast the retained modal coefficients using either autoregressive or ARIMA models.

Table 6 presents the equatorial Pacific test results. Anomaly persistence remains close to standard persistence, whereas EOF-AR and EOF-ARIMA yield larger errors. These results indicate that neither climatological anomaly propagation nor low-rank linear modal forecasting captures the nonlinear daily 3D variability as effectively as the recurrent base predictor and the proposed residual framework.

### 3.6. Ablation of Residual Learning and Full-Depth Gradient Guidance

**Figure 8:**
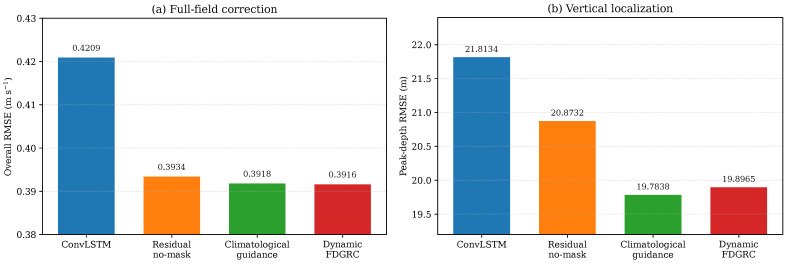
Mechanism ablation of the frozen residual framework. (**a**) Overall RMSE shows that residual learning supplies most of the full-field improvement. (**b**) Peak depth RMSE shows the additional contribution of climatological and dynamic gradient guidance to vertical localization. Lower values indicate better performance.

Table 7 separates the contributions of residual learning and vertical guidance. The full-depth no-mask residual model reduces the overall RMSE from 0.4209 to 0.3934 m/s, showing that a frozen-base residual branch accounts for most of the full-field improvement. Adding climatological or dynamic gradient guidance provides a further reduction to 0.3918 and 0.3916 m/s, respectively. The additional full-field gain is modest, but the guided variants improve the peak depth RMSE more clearly: 20.87 m for the no-mask model versus 19.78 m for the climatological mask and 19.89 m for FDGRC-Net. Thus, residual learning supplies the main global correction, while gradient guidance improves vertical localization. Figure 8 visualizes this decomposition: residual learning provides most of the overall RMSE reduction, whereas gradient guidance yields an additional improvement in peak-depth RMSE.

### 3.7. Multi-Seed Robustness

The climatological and dynamic full-depth variants were trained with seeds 42, 256, 571, and 1440. Each residual model was paired with the ConvLSTM-60d checkpoint trained using the same seed. Table 8 shows that both variants improve their corresponding base for every completed seed. The lower-overall-RMSE variant changes with initialization: the dynamic formulation is lower for seeds 42, 256, and 1440, whereas the climatological formulation is lower for seed 571. Consequently, the small difference between their mean full-field errors should not be interpreted as a statistically meaningful indication of superiority.

The mean ± standard deviation summary in Table 9 confirms that the two variants have effectively equivalent overall accuracy relative to the between-seed variation. The main repeatable distinction is the peak depth behavior: the dynamic formulation reduces the mean peak depth RMSE from 20.161±0.286 to 19.7527±0.0999 m and has a substantially smaller standard deviation. Because the seed count is limited, these values are used as descriptive robustness evidence rather than as the basis for a significance test.

### 3.8. Cross-Region Results in the Northern South China Sea

Both full-depth variants improve the retrained South China Sea ConvLSTM base under the same mask formula and hyperparameters used in the equatorial Pacific. As shown in Table 10, the dynamic formulation reduces the overall RMSE by 3.469% and the overall MAE from 0.1660 to 0.1625 m/s. Its overall RMSE difference from the climatological formulation is less than 0.0001 m/s, whereas its peak depth RMSE is approximately 1.01 m lower. The result supports the transferability of the full-depth localization mechanism without selecting a new regional correction band.

### 3.9. Representative Three-Dimensional Prediction Case

**Figure 9:**
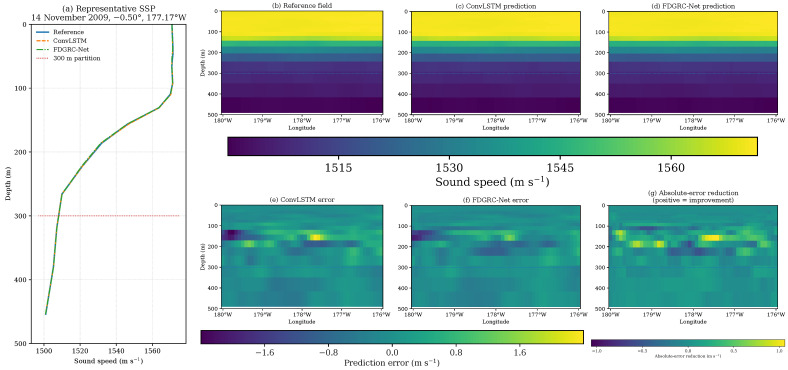
Representative next-day prediction for 14 November 2009, selected because its upper 300 m RMSE reduction equals the median over the 329 test dates. (**a**) Sound speed profile at the representative grid point. (**b**–**d**) Reference, ConvLSTM-60d, and FDGRC-Net depth–longitude sections. (**e**,**f**) Signed prediction errors. (**g**) Absolute error reduction, where positive values indicate improvement by FDGRC-Net. The dotted line marks the 300 m regional diagnostic partition.

Figure 9 presents the prediction for 14 November 2009. This date was selected objectively because its upper 300 m RMSE reduction, 0.0359 m/s, is equal to the median improvement over all 329 test dates. ConvLSTM-60d attains an upper 300 m RMSE of 0.4130 m/s on this date, whereas FDGRC-Net reduces it to 0.3771 m/s. The profile and depth–longitude sections show that both models reproduce the dominant 3D structure, while FDGRC-Net reduces localized errors around the upper-ocean gradient regime without introducing a deeper-field distortion.

### 3.10. Multi-Year Out-of-Time and ENSO Robustness

**Figure 10:**
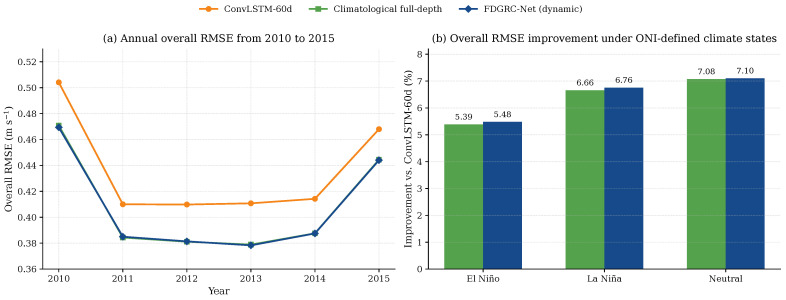
Frozen robustness evaluation beyond the development period. (**a**) Annual overall RMSE from 2010 to 2015. (**b**) Overall RMSE improvement relative to ConvLSTM-60d under ONI-defined El Niño, La Niña, and neutral conditions. Orange circles, green squares, and blue diamonds denote ConvLSTM-60d, the climatological full-depth model, and FDGRC-Net (dynamic), respectively.

The frozen 2000–2009 checkpoints are evaluated on 2010–2015 without retraining, normalization updates, hyperparameter selection, or mask updates. Figure 10 and Table 11 show that both full-depth variants outperform ConvLSTM-60d in every year. FDGRC-Net reduces the annual overall RMSE by 5.118–7.899%, with a daily improvement on 95.34–99.18% of target dates. The climatological variant follows the same pattern, with annual reductions of 5.039–7.765%. The lower-error full-depth variant changes across years: the dynamic model is marginally lower in 2010, 2013, 2014, and 2015, whereas the climatological variant is marginally lower in 2011 and 2012. Thus, the principal result is the stable advantage of full-depth gradient-guided correction over the frozen base, rather than large separation between the two mask formulations.

For FDGRC-Net, the 7-day moving-block bootstrap confidence intervals for the mean daily RMSE reduction remain strictly above zero in all six years; the lower bounds range from 0.0228 to 0.0316 m/s. The paired Wilcoxon checks lead to the same direction of effect in every year. The absolute prediction difficulty varies substantially, with the largest ConvLSTM RMSE values in 2010 and 2015, but no annual performance reversal is observed.

The ONI-stratified results in Table 12 show the same ordering under El Niño, La Niña, and neutral conditions. FDGRC-Net improves the overall RMSE by 5.479%, 6.755%, and 7.103%, respectively, and reduces the upper 300 m RMSE and peak depth RMSE in all three groups. The dynamic and climatological variants remain close, with the dynamic model being numerically lower in terms of the overall RMSE for each state. No performance reversal is observed across the three ONI-defined background conditions.

### 3.11. Observation-Based Consistency Against Argo Profiles

**Figure 11:**
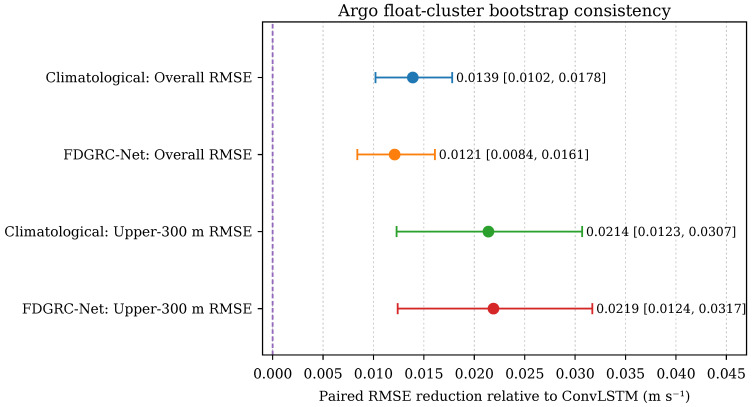
Observation-based paired consistency relative to ConvLSTM-60d. Points denote mean reductions in profile RMSE and horizontal intervals denote WMO float-level cluster bootstrap 95% confidence intervals. Positive values indicate closer agreement with the collocated Argo profiles.

The Argo workflow matched 531 GDAC files and decoded 728 inventory profiles. After quality control, 564 profiles remained; final temporal and spatial collocation yielded 521 profiles from 67 WMO floats over 461 unique prediction dates, with no download or decoding failures. Table 13 and Figure 11 summarize the primary bilinear collocation results. The collocated GLORYS12–Argo comparison provides a reference for the discrepancy between the reanalysis-derived target fields and the in situ profiles, with an overall RMSE of 1.496±0.505 m/s.

Relative to ConvLSTM-60d, both full-depth residual variants show small but consistent improvements in observation-based agreement. FDGRC-Net reduces the overall profile RMSE from 1.542 to 1.530 m/s and the upper 300 m RMSE from 2.827 to 2.805 m/s. The WMO float-level cluster bootstrap mean paired reductions are 0.0121 m/s for the overall RMSE (95% CI: [0.0084,0.0161]) and 0.0219 m/s for the upper 300 m RMSE (95% CI: [0.0124,0.0317]). The climatological variant produces corresponding reductions of 0.0139 m/s ([0.0102,0.0178]) and 0.0214 m/s ([0.0123,0.0307]). Both full-depth variants therefore improve the ConvLSTM forecast’s consistency with collocated observations, while their profile-level results remain close to each other.

### 3.12. Controlled Acoustic Propagation Diagnostic

**Figure 12:**
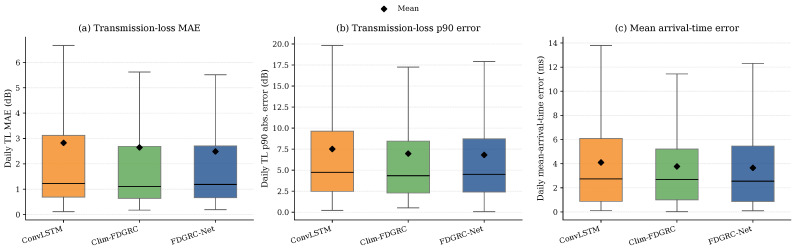
Daily acoustic error distributions over the 329-date 2009 test set: (**a**) transmission loss MAE, (**b**) 90th-percentile absolute transmission loss error, and (**c**) energy-weighted mean arrival time error. Boxes denote the interquartile range, whiskers denote the non-outlier range, and black diamonds denote daily means. Lower values indicate closer agreement with the Bellhop output generated from the GLORYS12-derived reference profile.

All 329 test dates were successfully evaluated for each of the three prediction models, yielding 987 model–date metric records and no Bellhop failures. Table 14 and Figure 12 summarize the 120 dB clipping results. FDGRC-Net reduces the mean daily TL MAE from 2.825±3.650 to 2.489±3.166 dB, an 11.91% reduction relative to ConvLSTM-60d. The improved-day fraction is 55.62%, and the 7-day moving-block bootstrap interval for the mean daily reduction is [0.139,0.571] dB, remaining above zero. The mean arrival time error decreases from 4.099±4.011 to 3.653±3.623 ms, with a corresponding improvement interval of [0.091,0.860] ms.

The climatological full-depth model has a lower mean TL MAE than ConvLSTM-60d, but its TL-MAE bootstrap interval [−0.026,0.392] dB crosses zero. Both full-depth variants reduce the mean 90th-percentile TL error, and the associated bootstrap intervals remain positive. FDGRC-Net also lowers the mean RMS delay spread error from 1.668 to 1.574 ms, although its interval crosses zero; this metric is therefore reported descriptively. The ordering of the mean TL MAE is unchanged under 100, 120, and 140 dB clipping, indicating that the principal conclusion is not specific to the selected clipping threshold. Together, these results show that full-depth residual correction can translate field-level prediction improvements into measurable reductions in transmission loss and arrival time errors under the controlled Bellhop configuration.

### 3.13. Computational Complexity Analysis

Computational complexity was measured on an NVIDIA GeForce RTX 3060 GPU (NVIDIA Corporation, Santa Clara, CA, USA) using CUDA runtime v11.8, a batch size of 1, an input size of 1×60×2×40×49×49, and an output size of 1×1×40×49×49. All models were evaluated in inference mode without gradient computation. Latency was measured after 20 warm-up runs over 100 synchronized CUDA trials and is reported as the mean ± standard deviation. Peak GPU memory was obtained after resetting the CUDA peak memory statistics. MACs denote multiply–accumulate operations, and FLOPs are reported using FLOPs=2×MACs. The ConvLSTM-60d parameter and MAC counts reproduce the previous complexity calculation, providing an internal consistency check. Table 15 summarizes the resulting parameter counts, operation counts, latency, and peak GPU memory usage for the representative methods.

FDGRC-Net includes the complete two-stage inference path: the ConvLSTM-60d background predictor, previous-day dynamic mask generation, and the residual correction network. Relative to ConvLSTM-60d, it adds 27,385 trainable parameters, while the FLOPs and mean latency increase by 5.0517% and 5.4188%, respectively. Peak GPU memory increases from 75.2983 to 98.6763 MB. The mask generator itself has no trainable parameters and operates only along the 40-level vertical profile, so most of the added cost comes from the residual Conv3D branch. TimeSformer3D-60d requires fewer FLOPs and lower latency than ConvLSTM-60d but yields higher prediction errors in the main comparison, indicating that the computational cost alone does not determine the forecasting accuracy. These measurements quantify the relative computational requirements on the tested GPU and do not constitute validation for embedded or operational deployment.

## 4. Discussion

### 4.1. Performance Gains from Residual Decomposition and Gradient-Guided Localization

FDGRC-Net improves a ConvLSTM-60d predictor rather than relying on a weak background forecast. On the 2009 equatorial Pacific test set, the framework reduces the overall RMSE from 0.4209 to 0.3916 m/s and the upper 300 m RMSE from 0.4733 to 0.4387 m/s. The corresponding reductions are 6.9610% and 7.3104%, respectively. Below 300 m, the RMSE also decreases from 0.2604 to 0.2488 m/s. The larger improvement in the upper ocean agrees with the diagnosed concentration of temperature gradients and prediction errors around thermocline-related depths, while the simultaneous deeper-field improvement shows that localized refinement does not compromise the broader 3D background field.

The mechanism ablation explains how these gains are obtained. The no-mask residual model recovers most of the full-field improvement, demonstrating that a frozen residual branch can effectively learn structured errors left by the ConvLSTM predictor. Adding climatological or dynamic gradient guidance further lowers the error and improves the peak depth accuracy. These results reveal complementary roles rather than competing explanations: residual decomposition provides the principal correction capacity, whereas full-depth gradient guidance directs this capacity toward vertically active layers. Freezing the first stage preserves the validated background solution and makes the added correction more stable and interpretable than jointly modifying the entire predictor.

The improvement is also achieved with a limited additional computational cost. Relative to ConvLSTM-60d, the complete FDGRC-Net inference path increases the FLOPs by 5.0517% and the mean latency by 5.4188%. Thus, the accuracy gain is not obtained by replacing the base model with a substantially larger end-to-end architecture but by adding a focused residual stage to address the remaining thermocline-related error structure.

### 4.2. Physical Value of Dynamic Full-Depth Guidance

The full-depth mask converts the previous-day regional temperature gradient profile into a continuous vertical weighting function over all 40 retained depth levels. This design avoids prescribing a fixed correction interval and allows the residual pathway to follow the vertical structure of the preceding ocean state. The development-period statistics support the physical basis of this design: all daily gradient peaks occur within the upper 300 m, the median peak depth is 155.8507 m, and the principal high-gradient range is approximately 109.7–222.5 m. The largest ConvLSTM error and the largest FDGRC-Net correction occur in the same thermocline-related depth range.

The climatological and dynamic variants achieve nearly identical mean full-field accuracy, which indicates that the development-period mean gradient provides an effective vertical prior. Nevertheless, the dynamic formulation adds state dependence without requiring a new regional depth band and yields lower and markedly more stable peak depth RMSEs across the matched-seed experiments. Its value therefore lies not only in the aggregate RMSE but also in adapting the correction profile to the immediately preceding ocean state and in providing a physically interpretable link between the temperature gradient structure and vertical prediction refinement.

### 4.3. Regional, Interannual, and Climate-State Robustness

The northern South China Sea has a shallower and structurally different upper-ocean gradient regime from the equatorial Pacific. Although the regional networks are retrained, the same full-depth mask formulation and hyperparameters improve the South China Sea ConvLSTM base, with FDGRC-Net reducing the overall RMSE by 3.4690% and lowering the peak depth RMSE from 29.2879 to 28.5967 m. This result shows that the proposed localization mechanism can remain effective across distinct thermocline structures without manually selecting a new correction band.

The frozen 2010–2015 evaluation provides a more demanding temporal test because no model parameters, normalization statistics, or climatological masks are updated after the 2000–2009 development stage. FDGRC-Net improves the overall RMSE in every evaluated year, with annual reductions of 5.12–7.90%. The same performance ordering is retained under El Niño, La Niña, and neutral conditions. Together with the matched-seed results, these experiments show that the improvement is sustained across initialization settings, regions, years, and large-scale climate backgrounds, rather than being confined to the original 2009 test split.

### 4.4. Observation-Based and Acoustic Relevance

The Argo comparison examines whether corrections learned from gridded GLORYS12 targets remain consistent with collocated in situ profiles. Although the resulting improvements are smaller than those measured against the gridded test fields, both full-depth variants reduce the additional profile error introduced by ConvLSTM-60d. For FDGRC-Net, the mean paired reductions are 0.0121 m/s for the overall RMSE and 0.0219 m/s for the upper 300 m RMSE, with positive WMO float-level cluster bootstrap intervals. Thus, the residual correction does not merely improve the numerical target used for training; it also preserves and slightly improves the agreement with the available observations.

The controlled Bellhop experiment further shows that field-level improvements can produce measurable acoustic benefits. Under identical propagation settings, FDGRC-Net reduces the mean TL MAE from 2.825 to 2.489 dB and the mean arrival time error from 4.099 to 3.653 ms. The TL-MAE ordering remains unchanged under three clipping thresholds. Although not every propagation metric improves to the same extent, the reductions in transmission loss and arrival time errors demonstrate that thermocline-aware 3D sound speed refinement can influence downstream acoustic calculations rather than remaining only a statistical improvement in field space.

### 4.5. Relation to Existing Prediction Approaches

The representative baselines indicate that daily 3D sound speed forecasting requires a different balance of spatial and temporal modeling from profile-level reconstruction or longer-term SSP prediction. Persistence and EOF-based methods do not capture the nonlinear daily evolution adequately, while the task-adapted CNN-BiLSTM, H-LSTM, STA-ConvLSTM, TimeSformer3D, and 3D U-Net models do not outperform ConvLSTM-60d in overall accuracy. In particular, the lower computational cost of TimeSformer3D does not translate into lower prediction errors, showing that architectural complexity or attention alone is insufficient for this task.

The main contribution of FDGRC-Net is therefore not simply a larger predictor. It separates background forecasting from residual refinement and makes the thermocline-related vertical structure an explicit modeling target. This addresses an aspect that is often obscured by profile-wide or field-wide losses: a model may reproduce the dominant 3D background while retaining concentrated errors around vertically sensitive layers. The proposed framework shows that diagnosing and correcting this residual structure can improve both the overall field accuracy and thermocline-related vertical metrics.

### 4.6. Limitations and Future Work

The present evaluation is substantially broader than a single regional test: it includes a second ocean region, six frozen out-of-time years, ONI-stratified conditions, collocated Argo profiles, and a controlled acoustic diagnostic. Nevertheless, the gridded training and primary test fields are derived from GLORYS12, and the regional experiments cover only the equatorial Pacific and northern South China Sea. Future studies should extend the evaluation to western boundary currents, shelf-break fronts, mesoscale eddies, and other ocean basins and should include alternative ocean products together with independent CTD, XCTD, and dedicated field observations. Such tests will clarify how the framework behaves under stronger horizontal gradients, rapidly varying fronts, and observational uncertainty.

The current dynamic mask is generated from a horizontally averaged regional temperature profile. Although this representation successfully captures the dominant thermocline-related structure and improves peak depth prediction, it cannot explicitly follow horizontal variations in thermocline depth within the 49×49 domain. A spatially varying 3D guidance field is therefore an important extension. Future work will also examine combinations of temperature gradients, sound speed gradients, predicted residual structures, and physical constraints to determine which representation most effectively describes thermocline variability.

Although the proposed framework consistently improves the ConvLSTM base, further improvement remains possible through alternative background predictors and correction strategies. The present work was motivated by the limited attention paid to thermocline-related localized errors in daily 3D sound speed prediction. Future research will systematically investigate additional recurrent, attention-based, and physics-guided approaches to better understand how the thermocline position, intensity, and horizontal variability affect the 3D forecasting accuracy and to develop stronger thermocline-aware correction methods.

Beyond the upper-ocean thermocline, the localized residual concept may be extended to other acoustically important structures. In particular, the daily 3D position and strength of the deep sound channel axis have not yet been modeled in the present framework. An adaptive axis-centered correction strategy could be developed and evaluated for deep-ocean prediction. The Bellhop analysis should also be expanded from the current controlled configuration to range-dependent environments, multiple frequencies and source–receiver geometries, ray-path and localization metrics, and field-calibrated acoustic observations.

## 5. Conclusions

This study proposes FDGRC-Net, a two-stage framework for next-day 3D ocean sound speed prediction that separates background evolution from thermocline-related residual refinement. A 3D ConvLSTM first produces a stable full-field forecast, after which a frozen residual branch uses a continuous mask derived from the previous-day full-depth temperature gradient profile to guide feature extraction, loss weighting, and output correction. This design introduces thermocline-aware vertical localization without prescribing fixed upper and lower correction boundaries.

On the 2009 equatorial Pacific test set, FDGRC-Net reduces the overall RMSE from 0.4209 to 0.3916 m/s, the overall MAE from 0.2302 to 0.2180 m/s, the upper 300 m RMSE from 0.4733 to 0.4387 m/s, and the peak depth RMSE from 21.8134 to 19.8965 m. The mechanism ablation shows that residual learning supplies most of the full-field gain, while gradient guidance provides additional vertical localization. Across four matched seeds, the dynamic formulation achieves a 0.3928±0.0066 m/s mean overall RMSE and produces lower and more stable peak depth errors than the climatological formulation. These gains require only a 5.0517% increase in FLOPs and a 5.4188% increase in mean latency relative to ConvLSTM-60d.

The same full-depth formulation improves the northern South China Sea experiment, all six frozen 2010–2015 annual evaluations, and the El Niño, La Niña, and neutral subsets. The Argo assessment shows that the correction remains consistent with collocated observations, while the Bellhop diagnostic demonstrates corresponding reductions in transmission loss and arrival time errors. Taken together, the results show that explicitly modeling the thermocline-related residual structure improves daily 3D sound speed prediction beyond a strong ConvLSTM background forecast.

Although further gains and broader validation remain possible, the present study addresses the limited consideration of thermocline-related error concentration in 3D forecasting by treating it as an explicit prediction target rather than relying only on field-wide metrics. Future work will extend this direction through spatially varying thermocline guidance, alternative prediction and correction architectures, independent field observations, additional ocean regimes, and adaptive modeling of the deep sound channel and downstream acoustic effects.

## Figures and Tables

**Table 1:** The 40 depth levels used in the experiments. Depths are in meters and are reported to four decimal places.

Index	Depth	Index	Depth	Index	Depth	Index	Depth
0	0.4940	10	15.8101	20	77.8539	30	453.9377
1	1.5414	11	18.4956	21	92.3261	31	541.0889
2	2.6457	12	21.5988	22	109.7293	32	643.5668
3	3.8195	13	25.2114	23	130.6660	33	763.3331
4	5.0782	14	29.4447	24	155.8507	34	902.3393
5	6.4406	15	34.4342	25	186.1256	35	1062.4399
6	7.9296	16	40.3441	26	222.4752	36	1245.2910
7	9.5730	17	47.3737	27	266.0403	37	1452.2510
8	11.4050	18	55.7643	28	318.1274	38	1684.2841
9	13.4671	19	65.8073	29	380.2130	39	1941.8929

**Table 2:** Exact chronological training, validation, and test split. Indices are zero-based and refer to target dates in the sorted daily sequence.

Subset	Target Indices	Target Date Range	Targets	Role
Training	365–2992	31 December 2000–11 March 2008	2628	Parameter estimation and calculation of training-only normalization statistics.
Validation	2993–3320	12 March 2008–2 February 2009	328	Checkpoint and configuration selection for the base predictor and residual correction variants.
Test	3321–3649	3 February 2009–28 December 2009	329	Held-out final evaluation and statistical analysis only.

**Table 3:** Task-adapted baseline implementations used for comparison.

Method	Original Focus	Adaptation in This Study	Purpose of Comparison
Standard Persistence	Classical reference forecast	The last observed 3D sound speed field is directly used as the next-day prediction.	Provides a non-learning lower reference.
Anomaly Persistence	Classical anomaly forecast	The latest anomaly relative to the training-period climatological background is propagated to the next day.	Tests whether separating the climatological background improves persistence.
EOF-AR	Reduced-order statistical forecast	A training-only EOF basis is used to represent the 3D fields, and retained modal coefficients are forecast with autoregressive models.	Tests linear low-rank temporal prediction of the full field.
EOF-ARIMA	Reduced-order statistical forecast	The same training-only EOF representation is combined with ARIMA forecasting of the retained modal coefficients.	Tests a more flexible classical time-series model in EOF space.
ConvLSTM-60d	Spatiotemporal sequence and sound speed prediction	Implemented as a 3D ConvLSTM predictor using daily 3D sound speed fields. Depth is treated as a spatial dimension rather than being flattened into channels.	Serves as the main deep temporal baseline and the frozen background predictor of FDGRC-Net.
CNN-BiLSTM-style	CNN-BiLSTM-Attention-based SSP prediction	The convolutional feature extraction and bidirectional temporal modeling ideas are retained, while the input and output layers are modified for daily 3D sound speed field prediction.	Tests whether profile-oriented CNN-BiLSTM modeling transfers to daily 3D field forecasting.
H-LSTM-style	Hierarchical few-shot SSP prediction	The depth-aware hierarchical recurrent modeling idea is retained, while the few-shot transfer learning setting is removed and the model is adapted to the same daily 3D input and output format.	Tests whether depth-aware recurrent modeling improves thermocline-related prediction.
STA-ConvLSTM-like	Multi-spatial-scale SSP prediction with spatiotemporal attention	The ConvLSTM and attention-based modeling idea is retained, while the original monthly SSP prediction setting is replaced by the daily 3D sound speed field prediction protocol.	Tests whether attention-enhanced ConvLSTM improves the base predictor.
TimeSformer3D-60d	Video modeling with divided space–time attention	The 60-day sequence is represented by 3D patch tokens and processed with divided temporal and spatial attention to predict the next-day 3D sound speed field.	Tests whether a video Transformer provides a stronger base predictor than recurrent convolutional modeling.
3D U-Net-60d	U-Net-style dense spatial prediction	A 3D encoder–decoder is trained using the 60-day historical sequence as stacked input features and outputs the next-day 3D sound speed field.	Tests whether a purely convolutional 3D encoder–decoder can replace recurrent temporal modeling.

**Table 4:** Window length ablation of ConvLSTM models.

Method	Input Window (Days)	Overall RMSE (m/s)	Comment
ConvLSTM-7d	7	0.4286	Short-window baseline
ConvLSTM-15d	15	0.4226	Window ablation
ConvLSTM-30d	30	0.4218	Window ablation
ConvLSTM-60d	60	**0.4209**	Selected base
ConvLSTM-90d	90	0.4231	Longer-window baseline
ConvLSTM-180d	180	0.4242	Long-window baseline
ConvLSTM-365d	365	0.5033	Long-window degradation

Note: Bold indicates the lowest overall RMSE.

**Table 5:** Main comparison on the equatorial Pacific test set. Upper 300 m and below 300 m denote z≤300 m and z>300 m, respectively.

Method	Type	Overall RMSE (m/s)	Overall MAE (m/s)	Upper 300 m RMSE (m/s)	Below 300 m RMSE (m/s)	Peak Depth RMSE (m)
Persistence	Reference baseline	0.5569	0.3010	0.6296	0.3298	22.1558
CNN-BiLSTM-style	Literature-style baseline	0.5518	0.3016	0.6221	0.3349	22.1530
H-LSTM-style	Literature-style baseline	0.5580	0.3026	0.6303	0.3324	22.1623
STA-ConvLSTM-like	Attention baseline	0.5568	0.3008	0.6294	0.3299	22.1790
TimeSformer3D-60d	Video Transformer baseline	0.4907	0.2739	0.5482	0.3187	21.6919
3D U-Net-60d	Spatial baseline	0.4445	0.2532	0.5021	0.2652	21.4648
ConvLSTM-60d	Deep temporal baseline	0.4209	0.2302	0.4733	0.2604	21.8134
FDGRC-Net (dynamic)	Proposed method	**0.3916**	**0.2180**	**0.4387**	**0.2488**	**19.8965**

Note: Bold indicates the best (lowest) value in each metric column.

**Table 6:** Additional classical statistical baselines on the equatorial Pacific test set. Lower RMSE indicates better performance.

Method	Forecasting Strategy	Overall RMSE (m/s)
Standard Persistence	Previous-day 3D field	0.5569
Anomaly Persistence	Previous-day anomaly relative to training-period climatology	0.5563
EOF-AR	Autoregressive forecast of training-only EOF coefficients	0.6587
EOF-ARIMA	ARIMA forecast of training-only EOF coefficients	0.8029
ConvLSTM-60d	Learned 3D recurrent forecast	0.4209
FDGRC-Net (dynamic)	Dynamic gradient-guided residual correction of the ConvLSTM forecast	**0.3916**

Note: Bold indicates the lowest overall RMSE.

**Table 7:** Ablation of residual learning and full-depth gradient guidance on the equatorial Pacific test set (seed 42).

Method	Vertical Guidance	Overall RMSE (m/s)	Overall MAE (m/s)	Peak Depth RMSE (m)	Improvement vs. Base
ConvLSTM-60d	None	0.4209	0.2302	21.8134	0.000%
Residual no-mask	None; full-depth residual only	0.3934	0.2194	20.8732	6.534%
Full-depth climatological	Development-period mean gradient	0.3918	0.2184	**19.7838**	6.907%
FDGRC-Net (dynamic)	Previous-day gradient	**0.3916**	**0.2180**	19.8965	**6.961%**

Note: Bold indicates the best value in each metric column (lower for error metrics and higher for improvement).

**Table 8:** Matched-seed results for the full-depth gradient variants.

Seed	Variant	Best Epoch	Overall RMSE (m/s)	Overall MAE (m/s)	Peak Depth RMSE (m)	Improvement vs. Same-Seed Base
42	Climatological	28	0.3918	0.2184	19.7838	6.907%
42	Dynamic	28	0.3916	0.2180	19.8965	6.961%
256	Climatological	29	0.4029	0.2246	20.3990	8.153%
256	Dynamic	29	0.4024	0.2246	19.7357	8.253%
571	Climatological	29	0.3866	0.2160	20.0958	8.455%
571	Dynamic	30	0.3876	0.2165	19.7107	8.212%
1440	Climatological	30	0.3897	0.2177	20.3660	7.241%
1440	Dynamic	29	0.3897	0.2182	19.6677	7.249%

**Table 9:** Four-seed mean ± standard deviation.

Variant	Overall RMSE (m/s)	Overall MAE (m/s)	Peak Depth RMSE (m)	Improvement vs. Same-Seed Base
Full-depth climatological	0.3928±0.0071	0.2192±0.0037	20.1611±0.2859	7.6889±0.7335%
FDGRC-Net (dynamic)	0.3928±0.0066	0.2193±0.0036	19.7527±0.0999	7.6685±0.6619%

**Table 10:** South China Sea test results using the unified full-depth evaluation protocol (seed 42).

Method	Vertical Guidance	Overall RMSE (m/s)	Overall MAE (m/s)	Peak Depth RMSE (m)	Improvement vs. Base
ConvLSTM-60d	None	0.2651	0.1660	29.2879	0.000%
Full-depth climatological	Development-period mean gradient	0.2559	0.1627	29.6023	3.467%
FDGRC-Net (dynamic)	Previous-day gradient	**0.2559**	**0.1625**	**28.5967**	**3.469%**

Note: Bold indicates the best value in each metric column (lower for error metrics and higher for improvement).

**Table 11:** Frozen annual out-of-time performance of FDGRC-Net from 2010 to 2015.

Year	Days	ConvLSTM Overall RMSE (m/s)	FDGRC Overall RMSE (m/s)	ConvLSTM Upper-300 RMSE (m/s)	FDGRC Upper-300 RMSE (m/s)	Overall Improvement	Daily Improved Fraction	95% CI of Daily Reduction (m/s)
2010	365	0.5041	0.4694	0.5736	0.5316	6.887%	97.53%	[0.0316, 0.0383]
2011	365	0.4100	0.3850	0.4572	0.4277	6.107%	97.81%	[0.0228, 0.0280]
2012	366	0.4098	0.3814	0.4580	0.4246	6.940%	99.18%	[0.0268, 0.0304]
2013	365	0.4107	0.3783	0.4605	0.4217	7.899%	99.18%	[0.0303, 0.0350]
2014	365	0.4142	0.3875	0.4657	0.4344	6.457%	98.90%	[0.0252, 0.0291]
2015	365	0.4680	0.4441	0.5295	0.5014	5.118%	95.34%	[0.0229, 0.0273]

**Table 12:** Frozen out-of-time performance stratified by ONI-defined ENSO state.

State	Days	ConvLSTM Overall RMSE (m/s)	Climatological Overall RMSE (m/s)	FDGRC Overall RMSE (m/s)	ConvLSTM Upper-300 RMSE (m/s)	FDGRC Upper-300 RMSE (m/s)	ConvLSTM Peak Depth RMSE (m)	FDGRC Peak Depth RMSE (m)	FDGRC Improvement
El Niño	547	0.4756	0.4500	0.4496	0.5394	0.5084	21.6819	19.7594	5.479%
La Niña	609	0.4393	0.4101	0.4096	0.4927	0.4573	15.2862	13.6257	6.755%
Neutral	1035	0.4153	0.3859	0.3858	0.4663	0.4315	21.9565	19.0967	7.103%

**Table 13:** Observation-based consistency against 521 collocated Argo profiles using bilinear horizontal collocation. Values are profile-level mean ± standard deviation.

Method	Profiles	Floats	Overall RMSE (m/s)	Overall MAE (m/s)	Upper-300 RMSE (m/s)	Bias (m/s)	Corr.
GLORYS12	521	67	1.496±0.505	0.998±0.286	2.727±0.894	−0.354	0.995
ConvLSTM-60d	521	67	1.542±0.535	1.021±0.299	2.827±0.952	−0.357	0.994
Full-depth climatological	521	67	1.528±0.533	1.010±0.299	2.805±0.944	−0.341	0.994
FDGRC-Net (dynamic)	521	67	1.530±0.530	1.014±0.297	2.805±0.943	−0.349	0.994

Note: Bold indicates the lowest value in each error-magnitude column.

**Table 14:** Controlled Bellhop diagnostic on the 2009 equatorial Pacific test set. Values are calculated over 329 paired dates.

Method	TL MAE Mean ± SD (dB)	TL MAE Median [IQR] (dB)	TL p90 Mean ± SD (dB)	Mean Arrival Error Mean ± SD (ms)	RMS Delay Spread Mean ± SD (ms)	TL-MAE Reduction 95% CI (dB)
ConvLSTM-60d	2.825±3.650	1.225 [0.689, 3.121]	7.509±7.660	4.099±4.011	1.668±1.634	—
Full-depth climatological	2.645±3.505	**1.103 [0.636, 2.687]**	6.962±7.030	3.766±3.659	1.824±1.670	[−0.026,0.392]
FDGRC-Net (dynamic)	2.489±3.166	1.185 [0.663, 2.705]	6.804±6.784	3.653±3.623	1.574±1.536	[0.139,0.571]

Note: Bold indicates the best-performing value for each diagnostic.

**Table 15:** Computational complexity of the representative methods. Latency and peak GPU memory were measured on an NVIDIA GeForce RTX 3060 with batch size 1.

Method	Params	MACs	FLOPs/Sample	Latency Mean ± SD (ms/Sample)	Peak GPU Memory (MB)
Persistence	0	0	0	0.0000±0.0000	0.0000
CNN-BiLSTM-style	196,037	1,620,516,728	3,241,033,456	15.3385±0.3323	78.2007
H-LSTM-style	1,249,709	24,728,320	49,456,640	2.9761±0.1300	63.8784
STA-ConvLSTM-like	19,554	14,196,657,280	28,393,314,560	32.6450±0.2477	129.5674
TimeSformer3D-60d	142,757	5,570,382,720	11,140,765,440	15.5171±0.3800	107.1055
3D U-Net-60d	198,505	4,394,281,440	8,788,562,880	4.9128±0.2178	111.9614
ConvLSTM-60d	73,633	51,764,413,600	103,528,827,200	52.4488±0.4363	75.2983
FDGRC-Net	101,018	54,379,390,720	108,758,781,440	55.2909±0.3820	98.6763

## Data Availability

The GLORYS12V1 temperature and salinity fields are publicly available from the Copernicus Marine Service under product ID GLOBAL_MULTIYEAR_PHY_001_030. The Argo profiles are publicly available through the Argo Global Data Assembly Centres. The NOAA ONI record is publicly available from the Climate Prediction Center. The core implementation of the ConvLSTM backbone, FDGRC-Net residual correction module, full-depth dynamic gradient mask generator, sound speed calculation utility, and a representative configuration file is publicly available at https://github.com/xq245/fdgrc-net-reproducibility (accessed on 30 July 2026).

## Abbreviations

The following abbreviations are used in this manuscript:

SSP	Sound Speed Profile
RMSE	Root Mean Square Error
MAE	Mean Absolute Error
EOF	Empirical Orthogonal Function
AR	Autoregressive
ARIMA	Autoregressive Integrated Moving Average
ConvLSTM	Convolutional Long Short-Term Memory
FDGRC-Net	Full-Depth Dynamic Gradient-Guided Residual Correction Network
GLORYS	Global Ocean Reanalysis and Simulation
